# Multi-criteria assignment problems for optimising the emergency medical services (EMS), considering non-homogeneous speciality of the emergency departments and EMS crews

**DOI:** 10.1038/s41598-023-33831-7

**Published:** 2023-05-09

**Authors:** Mariusz Drabecki, Eugeniusz Toczyłowski, Krzysztof Pieńkosz, Grzegorz Honisz, Klaudia Kułak

**Affiliations:** 1 Institute of Control and Computation Engineering, Warsaw University of Technology, Warsaw, Poland; 2 Silesian Centre for Heart Deseases in Zabrze, Zabrze, Poland; 3 Order of Malta Poland, Maltese Medical Service, Katowice, Poland; 4 Faculty of Medicine, Lazarski University Warsaw, Warsaw, Poland

**Keywords:** Applied mathematics, Computational science, Computer science, Health care, Health services, Public health

## Abstract

Dispatching of the EMS crews (ambulances) to awaiting patients and then directing the patients, that are already onboard, to appropriate Emergency Departments (ED), is a nontrivial decision problem. In many emergency medical systems it is handled by the Medical Dispatcher using various strategies—sometimes preferring the closest unit. However, applying a wrong strategy may result in transferring acute-state patients, who require very specialised medical aid, to low-speciality EDs with insufficient treatment capabilities. Then, they would need to be re-transferred to referential units, prolonging substantially the time to receive treatment. In some cases such a delay might make the treatment less effective or even impossible. In this work we propose two multi-criteria mathematical optimisation problems—the first one allows us to calculate the ambulance-to-patient assignment, the second one—to establish the patient-to-hospital assignment. These problems not only take the time-to-support criterion into consideration but also optimise for the speciality of care received by each patient. The ED dispatching problem proposed allows both for direct transfers of patients to referential units and for re-transferring them from non-referential EDs. The performance of the proposed approach is tested in simulations with real-life emergency cases from the NEMSIS data set and compared with classic assignment strategies. The tests showed the proposed approach is able to produce better and more fit-for-purpose dispatching results than other strategies tested. Additionally, we propose a framework for embedding the proposed optimisation problems in the current EMS/ED dispatching process.

## Introduction

Dispatching of ambulances to patients and then, patients to appropriate emergency departments, is a multi-stage decision process. On one hand, in an emergency situation, the help must come as soon as possible. On the other hand, however, the service must be well-suited to patient’s conditions. Currently, various EMS dispatching strategies may be used: dispatching of the closest idle unit, maximisation of the overall coverage, or maximisation of the preparedness of the EMS system^1–3^. Most importantly, the strategy of dispatching the closest ambulance has been proven to be sub-optimal already in 1972 and further confirmed by other research works^4–6^.

What is more, it should be noted that in many EMS systems ambulances differ as per the levels of speciality they can offer to patients. One example is the Polish national emergency medical system^7^, where ambulances are differentiated basing on the speciality they provide to the patients. Namely the following types of EMS units exist:

- *Basic*—ambulance with at least 2 members of staff being paramedics or nurses,
- *Specialist*—ambulance with at least 3 members of staff, one of them being a *system* doctor,
- *HEMS*—helicopter emergency medical service, with at least 3 members of staff, one of them being a *system* doctor,
- *Collaborating units*—organisations which normally do not provide public EMS services, yet might be dispatched if required (e.g. Order of Malta Ambulance Corps Poland).

In many European countries the process of handling a medical emergency call is as follows: first a caller dials an emergency number. All over the European Union they can dial 112—European general emergency number. If 112 is reached, the call would usually be taken by a non-medical dispatcher, who serves as the first triager. When the non-medical dispatcher decides that the call is medically valid, they would transfer the call to a dedicated professional medical dispatcher. The medical dispatcher would then investigate the call further, triage it appropriately and take care of assigning an appropriate EMS unit, if deemed necessary. This medical dispatcher would then also help the ambulance crew to find an appropriate destination hospital. For example such a model is present in Austria and Germany. In this approach other services (e.g. fire brigade) have their own dispatchers, who would be handling the call requiring their support. However, the decision problems faced by those dispatchers are out of scope of this paper.

In some countries it is also possible to bypass the 112 number and contact the professional EMS medical dispatcher directly, via a dedicated number. Example of such countries are: Poland, Romania and France. There is also possible another, much less common operational model, where the call is completely handled by the non-medical 112 dispatcher. Such a model is present in Finland^8^.

When present in the process, the medical dispatcher must face a decision-making problem through making a trade-off between the time requirement for the ambulance to arrive, and the speciality the crew can offer to the patients. Often, this process can be facilitated by the use of dedicated Medical Priority Dispatch Software, which is discussed further in the paper. The software however, helps in triaging and categorising the calls but does not optimise directly for which exact unit (in terms of its callsign) is best to respond. Decisions made may impact further treatment possibilities. For instance, dispatching an ambulance with no possibilities to teletransmit the ECG to regional specialist centre for consultations may result in misdiagnosis of serious cardiac pathologies, including ST-elevation myocardial infarction (STEMI)^9^. Thus, in optimal decisions of the ambulance-to-patient dispatching it is required to take into account both time-to-arrival and speciality of units.

Once the EMS unit is at the site, the team deepens the diagnosis of the patient condition. Then, based on the results, further decision must be made to select the appropriate Emergency Department (ED) by taking into consideration both its speciality required for the patient and the estimated time-to-arrival. In Poland, emergency departments are part of the national medical emergency system^7^. Just like the ambulances, EDs also offer different levels of specialities—local EDs, regional specialist centres, trauma centres. In this work we will be referring to the two last types as *referential EDs* and the local one as *non-referential ED*. Similarly to assigning of ambulances to patients, the problem of identifying the correct ED for a given patient is a nontrivial decision-making process that requires establishing a trade-off between the proximity to the ED and the speciality needed in the patient’s condition. According to the Polish regulations, establishing of the ED, to which the patient is to be taken, results from joint collaboration of the dispatcher with the chief of the emergency medical team caring for the patient.

Some acute conditions require highly specialised quick treatment in a referential unit within given time from symptom onset. Some examples of those are: aortic dissection (to be treated as soon as possible), STEMI (most effective treatment within 90 min. of first medical contact) or massive pulmonary embolism (most effective treatment within 48 h of onset)^10–12^. For treatment to be effective, the patient must be transferred to the referential hospital—either directly from the scene or via re-transferring from a non-referential unit. Yet, re-transferring may add some important delays on the time-to-treatment, making further treatment difficult to be effective. Therefore, it is necessary to find an optimal patient-to-hospital assignment strategy taking into both speciality and time-to-treatment.

In this paper we propose a novel multi-criteria optimisation problems towards both ambulance-to-patient and patient-to-hospital assignment problems, that take into consideration objectives such as time and speciality of the offered emergency service. Time and speciality requirements are not uniform across acute-state patients and depend greatly on their medical condition. We take this fact into consideration in our optimisation problems by optimising for both time-to-support and for speciality received by each patient. This is done for each patient individually (on a per-patient basis). In that sense, we aim to design an ambulance-to-patient and patient-to-hospital optimal assignment tool, that would pinpoint the best currently possible dispatch decisions taking into consideration clinical conditions of the patients. The tool is intended to facilitate dispatcher’s decisions by providing them with recommendations.

Dispatch in the paper is understood as establishing the best possible assignment of precise ambulances to precise patients, and further precise EDs to these patients. It is done taking into consideration the current operational state of the EMS system (e.g. number of ambulances available, number of hospital beds available, time-to-arrival of a given ambulance to the patient or time to arrive at a destination hospital). This is in contrast to understanding dispatch as triage and categorisation of emergency calls, which is sometimes found in literature. The optimisation problems proposed in this work aim to improve the decision support system in helping the medical dispatcher in assigning ambulances to acute-condition patients and then the patients to emergency departments, that can efficiently treat patients’ conditions. The proposed problem allows also for re-referral of patients between non-referential and referential hospitals. What is more, in this paper we also propose an embedding framework of the problems proposed into the current dispatching decision-making process.

The goal of this paper is to show the importance of considering not only a single criterion (mostly time) in the optimisation of ambulance-to-patient and patient-to-hospital assignments, but also other criteria related to speciality a given unit offers in treating a given urgent medical condition. We also aim to show the importance of considering patients’ medical requirements on those criteria expressed in terms of aspirations/reservations in the optimisation process. The paper outlines that it is both technically possible, and medically desirable, to incorporate the speciality criteria in the optimisation of assignment of resources. The outcomes of our work can be used in combination with currently used call categorisation software (e.g. Medical Priority Dispatch System—MPDS) and with currently existing patient transport protocols. These can be used as input to the optimisation problems proposed, enhancing the ability of assigning the appropriate unit—both in terms of time and speciality criteria.

To achieve this goal we propose two multi-criteria mixed integer linear programming (MILP) optimisation problems for optimising EMS assignment decisions. The first problem proposed yields a Pareto-optimal ambulance-to-patient dispatch, basing on patients’ requirements on ambulances’ time-to-arrival and speciality offered. These requirements are established on a per-patient basis with respect to their clinical condition. The second problem proposed yields a Pareto-optimal patient-to-hospital assignment, which also takes into consideration all patients’ requirements on time-to-arrival and ED’s speciality, estimated based on their clinical condition.

### Clinical situation

To reduce the morbidity and mortality that can result from the acute phase of an illness or injury, it is essential that the ambulance response procedure is quickly ensured and that the patient is transported to the correct hospital, depending on the patient’s needs and the current capacity of the emergency medical services. To do this, the patient’s health condition and the maximum possible waiting time required to provide qualified medical first aid must be estimated^13^. The world’s leading causes of death include cardiovascular diseases. Research indicates that more than 4 million Europeans die each year for that reason. According to the research conducted in 2016–2017 in Katowice, Poland, the most common causes for Emergency Medical Service interventions were non-traumatic internal emergencies, which most often included: hypertension, atrial fibrillation, myocardial infarction, pulmonary edema, atrioventricular blocks, strokes, chronic obstructive pulmonary disease (COPD) and a diagnosis of bronchial asthma^14^. In addition, the most common medical emergencies include sudden cardiac arrest, which can be caused by hypoxia, cardiac tamponade, poisoning, ionic disturbances and shock. Symptoms such as abdominal pain, arm pain radiating to the jaw, unusual headache, severe bleeding, and confusion remain worrisome^15^.

As mentioned, cardiac arrhythmias and cardiovascular diseases are the most common reasons for the Emergency Medical Service interventions. Direct threats to life include acute coronary syndromes, pulmonary embolism or abdominal aortic aneurysm, which, if untreated, can lead to death in a short period of time. Cardiovascular diseases continue to be world’s leading causes of death, of which 50% are caused by ischaemic heart disease^16^. According to the Institute for Health Metrics and Evaluations data from 2017, 1.6 million people in Poland developed ischaemic heart disease. On the other hand, the data made available by the National Health Fund show that more than 85,000 acute coronary syndromes were recorded in Poland in 2021. Cases of acute coronary syndromes have also been reported, with nearly 67,000 myocardial infarctions^17^.

Acute coronary syndromes (ACS) are mainly caused by an imbalance between the myocardial oxygen demand and its supply. The cause of the oxygen limitation is most often the presence of atherosclerotic plaque in the coronary arteries, but there may also be the presence of cardiac arrhythmias, and complications after hemorrhagic shock. ACS include ST-elevation myocardial infarction (STEMI), non-ST-elevation myocardial infarction (NSTEMI) and unstable angina^18^. The main symptom with which patients visit the ED is sudden pain or chest tightness, usually localized retrosternally with radiation to the shoulders, angle of the jaw and elbows^19^. The diagnosis is based on the record of the received electrocardiogram (ECG), which should be performed within 10 minutes of the first contact with the health care system and on the basis of clinical symptoms. Currently, ambulances are equipped with an ECG recording machine, which allows for a quick diagnosis. If there is an ST-segment elevation, we diagnose STEMI; if there is a non-ST segment elevation, we should measure the level of troponins, elevated levels of which may indicate myocardial infarction. Once ST-segment elevation is recognized, the patient requires rapid reperfusion therapy according to the latest European guidelines or percutaneous coronary intervention (PCI)^20^. Patients diagnosed with myocardial infarction should be transported by the Emergency Medical Service to a PCI-capable facility as soon as possible. For a patient not capable of primary PCI, fibrinolytic therapy should be started within less than 10 min. Current recommendations say that the patient should be transported to the nearest hemodynamics centre on 24-h duty, and not to the nearest hospital. When a patient with ST-segment elevation MI (STEMI) arrives at a non-ICU hospital, he or she should be immediately transported to an invasive cardiology unit^21^. A patient presenting to a hospital where PCI can be performed should receive treatment within no more than 60–90 min if fibrinolytic treatment fails, however, the maximum delay from STEMI diagnosis to reperfusion during PCI according to the Polish cardiac society is 120 min if a primary PCI strategy is chosen instead of fibrinolytic treatment. When immediate PCI is not possible, pharmacotherapy with invasive treatment should be considered, where coronary angiography is performed within 24 h^22^.

Apart from the above, nearly 5% of patients arriving in the ED are those with neurological symptoms. Sang-Beom et al. in their research distinguished a significant predominance of patients with stroke symptoms, epileptic seizures and status epilepticus among neurological emergencies. Among strokes, 80–90% of cases are patients with ischaemic stroke due to embolism or extra-cerebral vascular pathology, and misdiagnosis has been associated with increased mortality rates^23^. Ischaemic stroke is the second most common cause of death and long-term disability of adults worldwide, and the incidence of that disease increases with age. Fibrinolytic therapy is an effective treatment for stroke patients and the therapeutic window for intravenous tissue-type plasminogen activator therapy is 3–4.5 h from the onset of the first symptoms; however, only about 25% of patients on the ward receive thrombolytic treatment within the indicated time window^24,25^. Diagnosis and implementation of treatment of patients with symptoms of acute central nervous system injury determines the effectiveness of planned therapy, but often patients arrive at an intermediate hospital that does not have a stroke unit or lacks diagnostic and therapeutic capabilities, which delays the timing of thrombolysis. In order not to delay the therapeutic window, the emergency team should notify the stroke unit staff to reduce the occurrence of in-hospital delays, while inexperienced and unequipped centres for neuroimaging in the treatment of stroke patients have an indication to use remote consultation with reference centres^26^. As a result, only appropriate hospitals can provide treatment for stroke patients.

The introduction of intravenous thrombolysis with recombinant tissue-type plasminogen activator (rtPA, alteplase) to treat acute ischaemic stroke required a revolution in the organisation of stroke care. Recognition that “time is brain” drove effective public and prehospital awareness campaigns, such as the “Face, Arm, Speech, Time” (FAST) test^27^ and rapid prehospital triage to designated centres.

The organisation of stroke care depends upon local geography, but the implementation of dedicated acute stroke pathways varies widely. Comprehensive stroke centres provide all aspects of acute stroke care. Triage of patients eligible for endovascular thrombectomy directly to a comprehensive stroke centre (the “mothership” model) may improve the likelihood of good outcome, even if other hospitals are closer. Primary stroke centres are usually smaller centres that initiate intravenous thrombolysis and transfer patients eligible for endovascular thrombectomy to a comprehensive stroke centre, the so-called “drip-and-ship” model^28^. The key aspect of any stroke service model is that patients can access specialist expertise, neuroimaging and stroke unit care without delay^29^.

Worldwide, there exist accepted guidelines and dedicated protocols for the treatment of patients in life-threatening conditions and their transfer to dedicated centres. The European Resuscitation Council’s 2021 guidelines, indicate that a patient suffering from a cardiac arrest should be transported to a dedicated centre for treatment of reversible causes of cardiac arrest, basing on local guidelines^30^. Local guidelines are then developed for many locations. For example, in the US state-wide local transport protocols have been developed. These are present for instance in: Alabama^31^ and Delaware^32^. They are briefly discussed in this section.

In Delaware guidelines for patients diagnosed with ST-segment elevation myocardial infarction are based on the same indications to transport the patient as soon as possible to a facility capable of performing percutaneous coronary intervention PCI with concomitant pharmacological treatment. For paediatric patients, the guidelines point to the notion of effective chest compressions followed by transporting the paediatric patient from the scene to an ECMO-equipped facility as quickly as possible. Similarly, the state of Alabama has also adopted a protocol for bypassing primary care hospitals for patients with acute coronary syndromes and myocardial infarction with STEMI to hospitals with an accessible catheterization (PCI) laboratory.

Let us now consider guidelines for stroke patients. Delaware recommends to immediately transfer a stroke patient to the nearest specialised stroke center certified by the state of Delaware. To this end, criteria were adopted for VAN (Vision, aphasia, neglect) negative and LKW (last known well) patients with a time when they were last seen without stroke symptoms of less than 4.5 h, admission to the nearest specialized stroke center should be considered. For VAN positive and LKW patients more than 4.5 h, transport of the patient directly to a certified thrombectomy center should be considered. Similarly, the same procedures are adopted for stroke patients in Alabama.

Apart from cardiac and stroke cases, guidelines on bypassing the local facility also exist for trauma and burn patients. Patients assessed with the Glasgow Coma Score < 13 and low systolic pressure and respiratory count < 13 should be transported first to a highly specialised centre. It is also advised in Delaware that in case of an obvious injury, the patient is transported to the highest-level trauma centre. Detailed list of obvious injuries can be found in Ref.^32^. Similar guidelines on trauma handling are also found in the Alabama protocol. However, the protocol requires that the patient is diverted to the closest ED in case of: loss of airway, haemodynamic instability with no vascular access and external uncontrolled bleeding.

When it comes to burns, patients are required to be transported to a burn centre bypassing the nearest centre  basing on the percentage of burn area and on whether respiratory burns occurred. Assessment of whether a given patient is to be transported to a burn centre can be made using the *rule of nines*, also given in the protocols.

There are many emergency conditions that can lead to death. Hence, it is crucial to take action in the pre-hospital setting when transporting the patient to the hospital. Many of the acute conditions have a therapeutic window, i.e. a maximum time to implement therapy from the time of the first worrying symptoms. Delaying appropriate medical care in a specialised unit, in a serious condition practically does not guarantee survival. If a patient is transported to a hospital that has no specialised equipment and personnel, we delay the time to provide treatment at the cost of transporting the patient to a specialised centre.

### Literature review

Organising, operating and forecasting of Emergency Medical Services is a topic of extensive research. Computer-based systems might help in making well-suited, timely decisions to support operations of the whole EMS system, e.g. in assigning of ambulances to calls, assigning ambulances to EDs, ambulance routing, medical documentation handling or patient drop-off procedures and in notifications of staff required to handle a given emergency^33,34^.

Within that field, substantial number of research works focusing on the use of operational research (OR) methods for this purpose have been published. Authors of Ref.^35^ identified that researchers focus on applying OR in the following problems of EMS organisation: location of ambulances with their further relocation, dispatching and routing of ambulances, interplay of EMS with general health system as well as forecasting of calls and availability and crew scheduling. They also note that an important research area is development of simulation/validation tools. These observations have been backed up by the authors of another review paper^2^, who underlined also the necessity of staff hiring and fleet operations optimisation and of Ref.^1^ who reviewed the problems in EMS logistics.

Some interesting usages of OR models for emergency medical system planning are given in Refs.^36–39^, some also investigating fairness measures^40^. A number of significant papers have also been published in the field of forecasting^41^ and in management of patients once in ED or hospital^42–44^. Those however are not directly linked to the scope of this paper and thus are given only as a reference for an interested reader.

From statement perspective this paper builds on ambulance dispatch/allocation/routing problems. These problems have been of significant research interest. Jangtenberg with co-authors studied the dispatching of ambulances as applied to the Dutch practice^6,45^. Not only did they propose a new dispatch strategy outperforming the *closest idle*, but further proposed a benchmark model for offline optimal dispatching of ambulances. EMS dispatching taking into consideration equity call prioritisation was studied in Refs.^46,47^, where Enayati et al. focused also on simultaneous optimal location of ambulances. The notion of simultaneous optimisation of dispatch and location of ambulances was also applied in Ref.^48^. Authors depicted on the example of EMS data from Portugal that using OR tools with more advanced dispatch strategies can give better results than doing this by hand under *closest idle* criterion. Relocation optimisation and dispatch policies was also studied by Siong Lim et al.^49^, who reviewed dynamic ambulance relocation models from the perspective of dispatch policies. Their paper presents also a comparison of different EMS dispatch policies. Boutilier et al.^50^ however proposed to combine optimisation of location and routing of ambulances in the city of Dhaka, Bangladesh.

Interesting notion in dispatch optimisation is integration of considering different types (specialities) of ambulances^51–53^, i.e. *(ALS)*—Advance Life Support and *(BLS)*—Basic Life Support which are assigned to emergency calls basing on case severity. Knight et al.^54^ assess the severity with means of survival probability functions and operate the EMS system in order to maximise their expected value. As shown by Stout et al.^55^ , the fact of operating an all-ALS EMS system it is possible to reduce the complexity of triaging of calls and of defining what sort of unit should respond. What is more, in such systems there is no possible need of secondary triage on-scene (e.g. calling a different ambulance type for support). This however comes at the cost of possible prolongation of time-to-arrival and of dilution of certain paramedic skills. The latter is specifically important, since according to Stout et al., in only 10% of calls ALS skills are required.

When describing current state of the art in ambulance dispatching, we should mention the general Emergency Medical Dispatch software, and specifically the Medical Priority Dispatch System (MPDS). It is a software system, which aims to categorise emergency medical calls into numerical complaint-based categories and to assign them a given handling priority. The system provides the dispatcher with detailed questions, which are then asked to the caller. Basing on their answers the system categorises the call, assigns the handling priority. Then, the calls can have a sub-group and a modifier assigned to help responders in knowing the details of the case they are to deal with. The category, priority, sub-group and modifier together form the so-called MPDS determinant^56^. The MPDS is widely used across the world and in Europe itself for triage and categorisation of the calls^57^. It has been proven that the use of MPDS system has high sensitivity but moderate to low specificity in sending appropriate units to patients requiring ALS^58,59^. Despite this problem, Dong et al. showed that the use of an *optimised* version of MPDS in China led to an increased diagnosis consistency of the Acute Coronary Syndrome and reduced the call-to-patient arrival time^60^.

The classical version of the tool however, stops at categorising the calls, and not naming (in terms of exact callsign) the best unit to respond^61^. Since optimisation methods look at identifying the best possible decisions, combining them with MPDS may be a good idea. One could first categorise the call using MPDS and then find the best exact ambulance which should respond to the call via mathematical optimisation. Similar approach was proposed in Ref.^47^ , where authors perform multicriteria ambulance assignment (dispatch) optimisation considering different levels of priority of the emergency calls received. Although they do not state that the priorities are assigned using MPDS, one can easily deduct that MPDS could be a good candidate to perform this task.

After the EMS crew finishes stabilising the condition of the patient, the correct emergency department is to be identified. These problems have been studied in literature as well, mostly as ambulance routing or allocation problems. Talarico et al.^62^ investigated routing of ambulances transporting patients with different levels of acuity, yet they have not distinguished EDs basing on speciality they can offer to patients. This has been included as an additional criterion through weighted sum scalarisation in Ref.^63^. ED competence in ambulance allocation optimisation considering possible ED overcrowding was also included by Acuna et al.^64^. The authors have considered the speciality through constraints in the optimisation problem. An important contribution in the field of emergent cases assignment to EDs was given by Leo et al.^65^, where the authors included both speciality of units (as additional criterion, with weighted sum scalarisation) combined with the ED workload management.

From medical point of view, many patient transport protocols have been developed. These documents give guidelines to the responding teams on where to transport a given patient. Some examples of those are given for Alabama^31^ and for Delaware^32^. They give information on where and how to transport a given patient, basing on certain clinical criteria. For example, in Alabama it is recommended that the ambulance crew *considers* transporting a patient with STEMI to a hospital with catheterisation lab available. Yet, if the ambulance crew is unsure of the appropriate destination hospital, Online Medical Directors (OLMD) should be contacted for support. Similarly, in Delaware such a patient should be transported *when practical* to a PCI-capable facility, bypassing the closest hospital. A little bit more strict are the Polish Emergency Medical System plans, established for each of the 16 Polish voivodeships. As an example—in the Swietokrzyskie voivodeship exact addresses of hospitals capable of performing a given emergency medical procedures are named. The plan leaves choosing the most appropriate unit for a given patient X to the joint discretion of the medical dispatcher and of the chief of the medical team^66^.

Unfortunately not for all conditions such protocols exist, and not everywhere they were established. The authors of Ref.^67^ outlined that 78% of US states had implemented EMS triage and destination plans for trauma, around 33% for burns, stroke, and STEMI, while only 10% for cardiac arrest. This is in line with further findings of Authors of Ref.^68^ , who identified only 16 states with specific transport protocols for patients with stroke caused by large vessel occlusion (LVO). What is more, even if protocols are well adopted with dedicated nation-wide patient-care networks established, misdirection of patients can also happen. This is reported for European countries when referring to STEMI patients, for whom quick intervention in a PCI-capable hospital is crucial to reduce the mortality^69,70^. What is more, the protocols themselves provide guidelines on when to bypass the closest ED and transport the patient directly to a referential unit. In that sense, they do not assign a given ED (in terms of its exact address) to a given, precised patient X. Neither do they take into consideration the current operational state of the EMS system, e.g. in terms of current availability of hospital beds. That is why these protocols should be considered as input to optimisation procedures, which take care of assigning a very precise hospital to a given patient in urgency.

Our literature review outlined that, there exist some currently used interesting dispatch systems (MPDS) and EMS transport protocols. Dispatch systems however, focus mostly on performing the triage of calls and assigning a given priority to them. They do not perform the dispatch as understood by the OR community, i.e. do not give exact information on which unit (identified through its callsign) is best to respond to a given emergency. When it comes to EMS transport protocols, they give guidelines on with what sort of emergency should the ambulance crew consider taking the patient to a specialised ED. The protocols do not tell exactly that a given patient X is to be taken to the hospital Y, considering current operational state of the complete EMS system. These systems and protocols can integrate well with optimisation techniques. They can act as an input guidelines—by either estimating the priority of the call or by setting standards on what speciality should the destination hospital offer to a patient suffering from a specific medical condition. Then, taking this medical input, operational research (OR) techniques can be applied to determine and assign the currently best unit to respond to an emergency (either ambulance or ED). Our paper intends to fill this gap, by combining OR methods which allow for assigning exact units to exact patients in a Pareto-optimal way. This is done considering clinical condition of the patients and the current operational state of the EMS system.

Despite the fact that OR in EMS organisation is a topic of extensive research, the majority of papers mostly consider the time criterion in the ambulance-to-patient and patient-to-hospital dispatch. There exist, however some notable research works that include also the speciality levels of ambulances or EDs. From what we have found, it is mostly included in the optimisation problems as constraints or criterion with weighted sum scalarisation. We believe that inclusion of speciality in the form of constraints might greatly restrict the feasible set of the problem, and in some situations even make the dispatch infeasible. When it comes to weighted sum scalarisation however, we believe that assignment of appropriate weights to criteria might be a nontrivial task, especially for a medical dispatcher, who is not an expert in OR. Thus, this scalarisation might not be the easiest to be applied. What is more, to the best of our knowledge, we have not identified any paper that considered possible re-referrals of patients between a unit with lower speciality and the one with its higher level. In that sense our paper intends to fill the gap identified, as well as applies the Reference Point Method scalarisation, which we believe is well-suited for applications in *services of general interest*.

## Proposed approach

Assessing acute-condition patient’s emergency treatment needs vary depending on the stage of emergency medical services dispatching stage. First, the patient’s condition is assessed by the medical dispatcher basing on the symptoms observed by the caller and further medical interview performed by the dispatcher. This activity is usually facilitated by using the MPDS, which provides the dispatcher with a structured questionnaire which depends on the complaint given by the caller. Based on the information collected, the dispatcher sends an ambulance basing on the estimate of the patient’s condition. Then, Emergency Medical Service (EMS) crew is dispatched to site. Once arrived, the medics deepen the diagnostics and are able to professionally asses patient’s condition. Therefore, the dispatcher’s understanding of the patient’s condition varies depending on the stage of the dispatching process.

Having the changing nature of information on patient’s condition in mind, we propose to divide the overall dispatching problem of allocating both EMS units to patients and patients to Emergency Departments (EDs) into two distinct multi-criteria optimisation problems, i.e.

- *EMS Dispatching Problem* (P1)—problem of assigning adequate ambulances (EMS) to patients, by taking into account initial patients’ conditions given by the caller,
- *ED Dispatching Problem* (P2)—problem of assigning patients to adequate emergency departments by taking into account more actual patients’ conditions.

In this work we focus on acute cardiac conditions. Thus, in the remainder of the paper, whenever we refer to the *level speciality* it means specifically cardiological speciality. Yet, the approach and following formulations are general enough to be used directly whenever referring to any other possible medical emergency and speciality in treating any other condition.

In the proposed approach we specifically consider speciality of both EMSes and EDs. For modelling purposes, let us assume that the level of speciality is given by a real number

1 $$\begin{aligned} s \in [0;1], \end{aligned}$$

where $s=0$ means no cardiological speciality offered at all and $s=1$ means the best cardiological unit in the area. While taking the example of EMS dispatching those two extreme values could mean for example a taxi ($s = 0$) and a mobile intensive care unit ($s = 1$)^71^. Similarly, in the ED dispatching problem $s = 0$ could mean a general practice nurse office and $s = 1$ super specialised cardiology hospital. It is worth mentioning that we deliberately decide to model the level of speciality as a real number in the interval given in (1), rather than by a set of discrete choices as in Polish legislation (eg. ambulances *P,S,HEMS*). This is to better model varieties in speciality taking into consideration for instance different equipment onboard the ambulances or differences in crew’s experience in treating cardiac condition.

### EMS dispatching problem (P1)

In this section we propose a multi-criteria mixed-integer linear programme (MILP) optimisation model for assigning ambulances to patients, taking into account both speciality of the unit dispatched and time-to-arrival. The model of EMS Dispatching Problem is given in (2)–(6).

2 $$\begin{aligned} \text {max} \quad [s_1^1,-t_1^1,...,s_p^1, -t_p^1] \quad p = 1,2,..., |\mathcal {P} |, \end{aligned}$$

subject to

3 $$\begin{aligned}{} & {} s_p^1 = \sum _{a \in \mathcal {A}} s_a \ y_p^a \qquad \forall p \in \mathcal {P}, \end{aligned}$$

4 $$\begin{aligned}{} & {} t_p^1 = \sum _{a \in \mathcal {A}} t_p^a \ y_p^a \qquad \forall p \in \mathcal {P}, \end{aligned}$$

5 $$\begin{aligned}{} & {} \sum _{a \in \mathcal {A}} y_p^a =1 \qquad \forall p \in \mathcal {P}, \end{aligned}$$

6 $$\begin{aligned}{} & {} y_p^a \in \{0;1\} \qquad \forall p\in \mathcal {P}, \forall a \in \mathcal {A}. \end{aligned}$$

where

- $s_p^1$—speciality received by patient *p* through EMS dispatch.
- $t_p^1$—time it takes for dispatched ambulance to reach patient *p*.
- $s_a$—speciality level of ambulance *a*, $s_a \in [0,1]$
- $t_p^a$—time needed to reach patient *p* by ambulance *a*.
- $\mathcal {A}$—set of available ambulances.
- $\mathcal {P}$—set of patients needing support.
- $y_p^a$—binary variable describing assignment of ambulance *a* to patient *p*.

The problem by design is to make dispatcher’s decisions easier on choosing assignment of available ambulances to patients after receiving the emergency calls. Therefore, we assume that the patients are known a priori and that the number of calls is lower that the number of available ambulances. In the case when new emergency call appears when no ambulance is available, it should be handled later on, after some ambulances become idle. However, it is possible to extend the decision model by including queuing theory constraints, as proposed in Ref.^47^. This is deliberately omitted in this work, since our goal is to outline the importance and performance of the multi-criteria strategy, as opposed to standard single-criterion strategies. Additionally, we aim in the paper to show the importance of considering patients’ medical requirements in the optimisation process.

### ED dispatching problem (P2)

This section gives the multi-criteria MILP formulation of ED Dispatching Problem in (7)–(20). It is to be solved in the second step, after solving the EMS Problem, once the initial assessment made through the medical interview has been adjusted or confirmed by the emergency crew at site. ED Dispatching allows us to determine the dispatch of ambulances (with patients) to emergency departments. The problem gives the possibility to re-refer patients from a less specialised department to a more specialised one. The formulation takes into consideration the fact that admitting the patient first to a non-referential hospital and then to a referential one may boost the level of speciality received by the patient. This is due to the fact that some pre-treatment might be given to the patient in the non-referential unit. The factor by which the pre-treatment participates in total treatment is given by the arbitrary parameter $\eta _1$.

7 $$\begin{aligned} \text {max} \quad [s_1^2,-t_1^2,...,s_p^2, -t_p^2] \quad p = 1,2,..., |\mathcal {P} |, \end{aligned}$$

subject to

8 $$\begin{aligned}{} {} s_p^2 &= \eta _1 \ \left( \sum _{h1 \in \mathcal {H}_{\text {not}}} s_{h1} \ y_p^{h1}\right) + (1-\eta _1) \ \left( \sum _{h1 \in \mathcal {H}_{\text {not}}} w_p^{h1} \ s_{h1}\right) \nonumber \\{} & {} \quad + \left( \sum _{h2 \in \mathcal {H}_{\text {ref}}} s_{h2} \ b_p^{h2}\right) + \left( \sum _{h2 \in \mathcal {H}_{\text {ref}}} s_{h2} \ u_{p,h1}^{h2}\right) \nonumber {} & {} \quad \forall p \in \mathcal {P}, \ \forall h1 \in \mathcal {H}_{\text {not}}, \ \forall h2 \in \mathcal {H}_{\text {ref}}, \end{aligned}$$

9 $$\begin{aligned}{} & {} w_p^{h1} \le y_p^{h1} \qquad \forall p \in \mathcal {P}, \ \forall h1 \in \mathcal {H}_{\text {not}}, \end{aligned}$$

10 $$\begin{aligned}{} & {} w_p^{h1} \le 1- \sum _{h2 \in \mathcal {H}_{\text {ref}}} u_{p,h1}^{h2} \qquad \forall p \in \mathcal {P}, \ \forall h1 \in \mathcal {H}_{\text {not}}, \end{aligned}$$

11 $$\begin{aligned}{} & {} w_p^{h1} \ge y_p^{h1}+ (1- \sum _{h2 \in \mathcal {H}_{\text {ref}}} u_{p,h1}^{h2}) -1 \qquad \forall p \in \mathcal {P}, \ \forall h1 \in \mathcal {H}_{\text {not}}, \end{aligned}$$

12 $$\begin{aligned}{} & {} t_p^2 = \sum _{h1 \in \mathcal {H}_{\text {not}} } t_p^{h1} \ y_p^{h1} + \sum _{h2 \in \mathcal {H}_{\text {ref}} } t_p^{h2} \ b_p^{h2} + \sum _{h1 \in \mathcal {H}_{\text {not}}} \sum _{h2 \in \mathcal {H}_{\text {ref}}} g_{h1,p}^{h2} \ u_{h1,p}^{h2} \qquad \forall p \in \mathcal {P}, \end{aligned}$$

13 $$\begin{aligned}{} & {} \sum _{p \in \mathcal {P}} y_p^{h1} \le \overline{H_{h1}} \qquad \forall h1 \in \mathcal {H}_{\text {not}}, \end{aligned}$$

14 $$\begin{aligned}{} & {} \sum _{p \in \mathcal {P}} b_p^{h2} + \sum _{p \in \mathcal {P}} \sum _{h1 \in \mathcal {H}_{\text {not}}} u_{h1,p}^{h2} \le \overline{H_{h2}} \qquad \forall h2 \in \mathcal {H}_{\text {ref}}, \end{aligned}$$

15 $$\begin{aligned}{} & {} u_{h1,p}^{h2} - y_{p}^{h1} \le 0 \qquad \forall p \in \mathcal {P}, \forall {h1} \in \mathcal {H}_{not}, \forall {h2} \in \mathcal {H}_{ref}, \end{aligned}$$

16 $$\begin{aligned}{} & {} \sum _{h_2 \in \mathcal {H}_{ref}} u_{h1,p}^{h2} \le 1 \qquad \forall p \in \mathcal {P}, \forall {h1} \in \mathcal {H}_{not}, \end{aligned}$$

17 $$\begin{aligned}{} & {} \sum _{h_1 \in \mathcal {H}_{not}} y_{p}^{h1} +\sum _{h_2 \in \mathcal {H}_{ref}} b_{p}^{h2} = 1 \qquad \forall p \in \mathcal {P}, \end{aligned}$$

18 $$\begin{aligned}{} & {} y_p^{h1} \in \{0;1\} \qquad \forall p\in \mathcal {P}, \forall h1 \in \mathcal {H}_{\text {not}}, \end{aligned}$$

19 $$\begin{aligned}{} & {} b_{p}^{h2} \in \{0;1\} \qquad \qquad \forall p\in \mathcal {P}, \forall h2 \in \mathcal {H}_{\text {ref}}, \end{aligned}$$

20 $$\begin{aligned}{} & {} u_{h1,p}^{h2} \in \{0;1\} \qquad \forall p\in \mathcal {P}, \forall h1 \in \mathcal {H}_{\text {not}}, \forall h2 \in \mathcal {H}_{\text {ref}}, \end{aligned}$$

where

- $s_p^2$—speciality received by patient *p* through ED dispatch.
- $t_p^2$—time it takes for patient *p* to reach final ED destination.
- $s_{h1/h2}$—speciality offered by ED $h1 \in \mathcal {H}_{not}$ or by $h2 \in \mathcal {H}_{ref}$.
- $\mathcal {H}_{ref}$, $\mathcal {H}_{not}$ — sets of available emergency departments—referential and non-referential respectively.
- $\eta _1$—factor by which patient is treated by the first emergency department, $\eta _1 \in [0;1]$.
- $t_p^{h1/h2}$—time needed to drive patient *p* to ED $h1 \in \mathcal {H}_{not}$ or to $h2 \in \mathcal {H}_{ref}$.
- $g_{h1,p}^{h2}$—time needed to re-refer patient *p* from non-referential ED $h1 \in \mathcal {H}_{not}$ to referential ED $h2 \in \mathcal {H}_{ref}$.
- $\overline{H_{h1/h2}}$ —maximum available capacity of emergency ED $h1 \in \mathcal {H}_{not}$ or by $h2 \in \mathcal {H}_{ref}$ at dispatch time.
- $y_p^{h1}$—binary variable describing assignment of non-referential hospital $h1 \in \mathcal {H}_{not}$ to ambulance transporting patient *p*.
- $b_p^{h2}$—binary variable describing assignment of referential hospital $h2 \in \mathcal {H}_{ref}$ to ambulance transporting patient *p*, direct transport to the referential hospital.
- $u_{h1,p}^{h2}$—binary variable describing re-referral of patient *p* from ED $h1 \in \mathcal {H}_{not}$ to ED $h2 \in \mathcal {H}_{ref}$.
- $w_p^{h1}$ —linearisation variable of binary product: $y_p^{h1} \ (1- \sum _{h2 \in \mathcal {H}_{\text {ref}}} u_{p,h1}^{h2})$.

Derivations of speciality and time-to-treatment delivered to the patient *p* are given in constraints (8) and (12). Constraints (9)–(11) ensure the linearisation of binary product of $w_p^{h1} = y_p^{h1} \ (1- \sum _{h2 \in \mathcal {H}_{\text {ref}}} u_{p,h1}^{h2})$, which is derived in order to get delivered speciality equal to $s_{h_1}, h_1 \in {H}_{not}$ if *p* is not re-referred to $h_2 \in {H}_{ref}$. If the re-referral happens, calculated speciality delivered will be equal to $\eta _1 s_{h_1} + s_{h_2}, \ h_1 \in {H}_{not}, h_2 \in {H}_{ref}$. Constraints (13) and (14) assure that current capacities of EDs are not violated. Constraint (15) assures that it is only possible to re-refer *p* from $h_1 \in {H}_{not}$ to $h_2 \in {H}_{ref}$, if *p* was first transported directly to $h_1$. Formulations (16) assures that *p* must be taken directly to exactly one of hospitals $h_1 \in {H}_{not}$ or $h_2 \in {H}_{ref}$ and (17) that *p* can be re-referred to maximum one $h_2 \in {H}_{ref}$, yet this is not mandatory.

### Embedding into current decision process

It is possible to embed the optimisation models given in “EMS Dispatching Problem (P1)” and “ED Dispatching Problem (P2)” sections and into standard decision process of the dispatcher, rather than completely re-organising it. Since both optimisation problems are multi-criteria, to solve them, decision-maker’s (DM’s) preferences towards all criteria should first be estimated^72^. In the proposed approach, criteria are associated with each patient’s medical condition and the dispatcher takes the role of the DM. Having the above in mind, we propose that preferences are given through estimation of *reservations* and *aspirations* towards all criteria. Mathematically, aspiration for criterion $f_i$ is the value the DM wants the $f_i$ to take and reservation—the value that is still acceptable for the criterion $f_i$, yet not best. This is explained in more details in “Scalarisation” section. In that way it is possible to reflect preferences as a direct function of patient’s condition, rather that through hard to understand and to explain weights. Assignment of weights could possibly disadvantage some patients and in that sense contradict with the fact that emergency medical systems is considered by the European Union as a *service of general interest*^73^.

**Figure 1:**
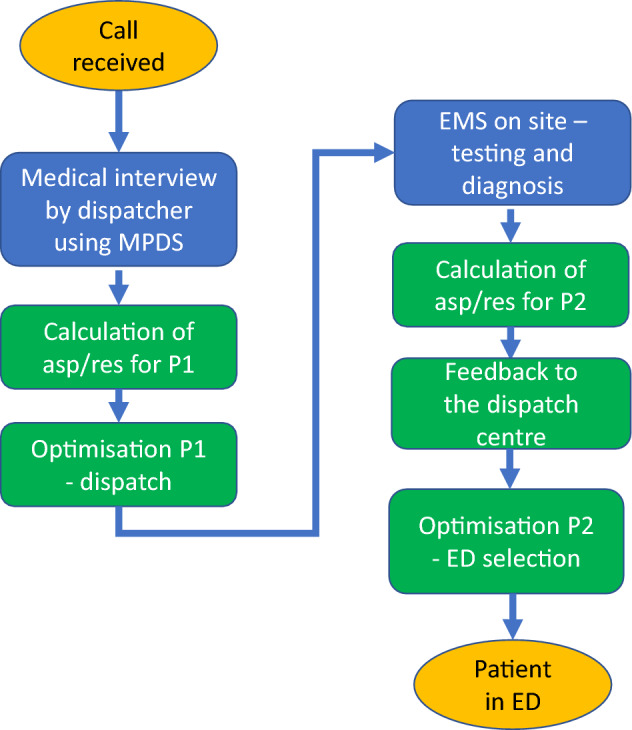
Proposed embedding framework in current decision process.

The integration schematic framework is shown in Fig. 1. The additions proposed in this paper are shown as green rectangles. Standard process elements are shown as blue rectangles and orange ellipses for starting and ending events.

The starting point of decision-making problem is receipt of an emergency call by the dispatcher. We propose to take advantage of the medical interview supported by MPDS. After some adjustments the MPDS (or similar system) could be leveraged to calculate aspirations/reservations for EMS Dispatching problem basing on the symptoms given by the caller. Once they are known, the optimisation takes place and optimal EMS crew is dispatched.

After that, we propose that after EMS’ arrival and additional diagnostics, the emergency team use their portable tablets/computers, such as the ones currently being part of the State Command Support System for the State Emergency Medical System—*SWD PRM*^74^. In this process the existing patient transport protocols can be embedded in the SWD PRM system to calculate aspirations and reservations basing diagnostics performed by the crew. Taking into considerations the aspiration and reservation values calculated, optimisation takes place in the dispatch centre in order to identify optimal emergency department for patient’s condition. The process stops when the patient arrives at the ED.

#### Example

Let us now demonstrate an example of how the proposed decision process can work in practice of EMS/ED dispatch. Note that to better show the decision-making process of our proposed solution in the example we calculate aspirations/reservations in a truly simplified way. In a real-life situation the dispatcher should ask many more detailed questions, possibly following the recommendations of MPDS. This would result in a much more granular way of calculating the values. The goal of this section is only to give the reader the feeling of the proposed solution could be put in action, hence the example is very simple and straight-forward. Let us consider the following situation:

1. *Medical dispatcher receives an emergency call. The caller gives following patients’ symptoms in the course of medical interview: acute chest pain radiating to the left arm, conscious, breathing.* Since symptoms can be significant for an acute myocardial infarction, this call is treated as urgent and as requiring specialist EMS care. Therefore, the interactive questionnaire (or modified MPDS) estimates the aspiration for EMS arrival at site ($a_{t_{EMS}}$) to 7 min. and the reservation for EMS arrival at site ($r_{t_{EMS}}$) to 14 min. Since possible teletransmission of ECG to the specialist centre might be required, aspiration towards EMS speciality ($a_{s_{EMS}}$) is estimated to 0.9 and reservation ($r_{s_{EMS}}$) to 0.7. Therefore for this particular patient we require an ambulance that would arrive in time shorter than 14 min and ideally in 7 min, offering the speciality greater than 0.7 and ideally 0.9.
2. *EMS is dispatched according to the preferences described in pt. 1 and arrives within 10 min. Once the crew arrives at scene they confirm the symptoms given by the caller. ECG with teletransmission to the on-duty cardiologist is performed, which reveals significant ST segment changes. Basing on them pre-hospital diagnosis of ST-elevation myocardial infarction was made.* STEMI is a condition that should be optimally treated in a highly specialised cardiologist centre within 120 min. Thus, the following aspirations and reservations towards time-to-arrival and speciality of the ED were calculated using EMS crew’s portable computer are: $a_{t_{ED}}=20, r_{t_{ED}} = 120, a_{s_{ED}}=0.9, r_{s_{ED}}=0.8$. Thus, it is required that this patient arrives at an ED with speciality greater than 0.8, ideally 0.9 and in time shorter than 120 min, ideally in 20 min. Please note that aspiration/reservation towards the time criterion were estimated such that they include the total time-to-arrival/time-to-treatment. These values are then fed back to the dispatch centre for optimal assignment of the Emergency Department. Once established, the EMS crew take the patient to the ED chosen.

The points above illustrate the idea how the process could be seen within a realistic example. Once the values of aspirations and reservations are calculated, the optimisation happens taking them as the decision model. Calculation of aspiration/reservation values for other patients is analogous to the case presented. More information on how this can happen is given in “Scalarisation” section.

Please note also that sometimes it may be possible to assign units by giving better final results than the value of the aspiration levels for some of the patients, and not worsening the results for the others. Such a dispatch will be selected by the optimiser.

## Scalarisation

Both proposed problems are multi-criteria MILP formulations. To solve them, proper problem scalarisation is needed. Since the criteria are associated with patients’ conditions, Pareto-optimality of the obtained result must always be ensured. Multi-criteria decision making is sometimes understood as as generating a list of Pareto-optimal (efficient/non-dominated) solutions and letting the DM decide on which solution they prefer^75^. Yet, the nature of the problems described is different. In the case given, we are interested in obtaining a dispatch that is feasible, Pareto-optimal and meets patients’ requirements on both speciality and time in shortest time possible. Only when the result is given fast the tool could help the dispatcher. Thus, listing many possible solutions and asking the dispatcher to check one after another is not required.

As described in “Embedding into current decision process” section we propose that DM’s preferences towards criteria are given as aspirations (desirable values that the criteria should take) and reservations (still acceptable values of criteria). In the remainder of the paper those values for a given criterion *i* will be referred to as $a_i$ and $r_i$ respectively. Scalarisation method that allows their direct integration is the *Reference Point Method* (RPM)^76^ with partial achievement function as introduced in Ref.^77^.

Those achievement functions could be viewed as a mapping of DM’s satisfaction on obtained values of criteria. Achievement functions are piece-wise linear and strictly increasing over the entire domain. Let $h_i$ be the value of partial achievement function for criterion $f_i$. It is negative and increases at a very steep slope until $f_i$ reaches $r_i$. Once $f_i =0$, $h_i$ also equals 0. Then, when $r_i<f_i\le a_i$ $h_i$ keeps increasing but at a more gentle slope until $f_i=a_i$, when $h_i$ takes the value of 1. Achievement function keeps increasing even further but at a very gentle slope. The formula for $h_i$ is given in (21).

21 $$\begin{aligned} h_i={\left\{ \begin{array}{ll} \gamma \frac{f_i-r_i}{a_i-r_i} \quad \text {for} \quad f_i \le r_i \\ \frac{f_i-r_i}{a_i-r_i} \quad \text {for} \quad r_i< f_i < a_i \\ \beta \frac{f_i-a_i}{a_i-r_i}+1 \quad \text {for} \quad a_i \le f_i \end{array}\right. }, \end{aligned}$$

where $\gamma$ and $\beta$ are arbitrarily taken constants such that $0< \beta< 1 < \gamma$. Under this assumption partial achievement function is strictly increasing and concave^75^. For instance $\beta$ may take order of magnitude of $10^{-3}$ and $\gamma$ of $10^3$.

The goal of RPM scalarisation is then to maximise the smallest achievement function over all criteria, with a very small component that assures Pareto-optimality of the result. The detailed description of the RPM as applied in this work, can be found in our previous paper^78^ or directly in the source references, i.e.^76,77^.

Let us now come back to the example given in “Example” section. The RPM scalarisation is mostly designed to maximise the smallest achievement function for all of the patients—taking into account both time and speciality. In other words, the optimiser will be trying to find a feasible and Pareto-optimal dispatch, such that obtained time and speciality criteria for all of the patients lands within the interval $[r_i,a_i]$. Of course this might not always be possible, yet it will be trying to get the best result possible for the most disadvantageous patient. Such a behaviour is desirable in dispatch of services of general interest, since it is assured that no-one is left behind in the dispatch process. What is more, the scalarisation is designed also in a way that the achievement functions keep increasing even after passing the value of aspiration. Thus, if possible, the resulting dispatch might be better than requested for some patients. This can only happen if the improvement for some patients does not make the dispatch worse for the others.

## Case study

We test the proposed approach in simulations. To make them more viable we took the real-life activations of the American EMS, from the 2020 National Emergency Medical Services Information System (NEMSIS) Public-Release Research Data set^79^. In this data set extensive information on situation of the system at the moment of the call and on health condition of the urgent patient is given. This includes both symptoms given by the caller to the dispatcher and diagnosis made by the EMS crew once arrived at scene.

For our analysis we took data on 41 patients with cardiac condition coming from the data set. For this we took the values of following atributes for each patient from the set of 41 considered

- primary symptom,
- provider’s primary impression,
- complaint reported by Dispatch to EMS (understood as symptoms given by the caller),
- flag if cardiac arrest happened,
- cardiac arrest etiology,
- first monitored arrest rhythm of the patient,
- reason why CPR stopped,
- end of EMS cardiac arrest event,
- age,
- possible injury,
- systolic BP,
- SpO2,
- respiratory rate,
- heart rate,
- ETCO2,
- pain scale score,
- ECG type,
- level of responsiveness (AVPU),
- stroke scale score,
- Glasgow coma scale,
- cardiac rhythm (ECG).

Detailed information on the attributes available in NEMSIS data set can be found in the NEMSIS Data Dictionary^80^.

For all 41 patients considered we arbitrarily assigned aspiration and reservation values towards both time and speciality for both problems considered (P1 and P2). This was done basing on expert knowledge assessment of the cases basing on attribute values given in the data set.

The optimisation problems considered were coded in Matlab using CVX, a package for specifying and solving convex programs^81,82^ and solved using Gurobi.

### Example of assigning aspirations/reservations

This section describes an example of assigning values of aspirations and reservations for the two problems considered (P1 and P2). For this we give the rationale for assigning those values to two cases of acute condition patients—Patient A and Patient B. Values of *selected* attributes from the list are given in Table 1 . Due to text length limitations, in this paper we deliberately show only selected attributes in this example, since not all of them are directly relevant in assigning the values of aspirations/reservations for these patients. One should remember that this activity is very case-specific and depending on the condition itself different vital parameters will be taken into account. What is more, all information regarding cardiac arrest are dropped since it did not occur in the discussed patients. However, the authors ask an interested reader who would like to get the full picture of all recorded parameters to reach out to them directly and those values will be provided.

**Table 1:** Example patients’ attributes.

Parameter	Patient A	Patient B
Primary symptom	Chest pain, unspecified	Chest pain, unspecified
Complaint reported by dispatch to EMS	Transfer/interfacility/palliative care	Heart problems
Age	67 years	47 years
Possible injury	No	No
Systolic BP	109	110
Heart rate	81	64
Respiratory rate	18	12
SpO2	96	100
ECG type	12 Lead-left sided	4 Lead
Level of responsiveness (AVPU)	Alert	Alert
Glasgow coma score	15	15
Cardiac rhythm	STEMI anterior ischemia	STEMI inferior ischemia

The estimated aspiration and reservation values for both problems (EMS Dispatching and ED Dispatching) for patients A and B are given in Table 2.

**Table 2:** Aspiration and reservation values of the exemplary patients.

	Patient A and Patient B
$a_{t_{EMS}}$ [min]	7
$r_{t_{EMS}}$ [min]	14
$a_{s_{EMS}}$	0.9
$r_{s_{EMS}}$	0.7
$a_{t_{ED}}$ [min]	20
$r_{t_{ED}}$ [min]	120
$a_{s_{ED}}$	0.9
$r_{s_{ED}}$	0.8

The primary symptom shown in both patients is chest pain, not related to any trauma. What is more, In Patient B the problem was assessed by the dispatcher as related to the heart. Patient A, however, was being transferred between facilities. Given the nature of the primary symptom, plus information in the complaint strict values for P1 speciality and time-to-arrival aspiration and reservation values were assigned. Since the case study presented is only an example of the performance of the multi-criteria method as opposed to other benchmarks with the same patients considered, we assigned the values of aspirations/reservations basing on the primary complaint. This was also done in that way due to the fact that not all important data are available in the NEMSIS data set for the patients studied. In a real-life situation the dispatcher should take into consideration more aspects before assigning the aspiration/reservations. This limitation does not impact the conclusions made, since we took the very same patients for all techniques considered.

As assessed by the EMS on site, both patients considered suffer from ST-segment elevation myocardial infarction (STEMI). Significance of total ischaemic time in the context of STEMI is very important. Prolonged total ischaemic time is a problem not specific to a certain geography or population, it exists across the world with varying degrees of intensity. Total ischaemic time strongly correlates as an independent predictor of major adverse cardiovascular events (MACE). Shorter ($<3$ h) total ischaemic time is associated with reduced risk of mortality.

One of underlying mechanism of increased mortality with prolongation of ischaemic time, is that infarct size significantly affects myocardial tissue and keeps on damaging with every passing second of ischaemic time. Prolonged total ischaemic time associates with higher mortality of STEMI patients in whom the recommended “door to balloon” is achieved. Hence, even with optimal reperfusion (primary PCI), prolonged ischaemic time may cause higher mortality and less myocardial salvage. Decrease in “door to balloon” time is unlikely to render the ultimate desired reduction in mortality after primary coronary angioplasty.

As given, the treatment is based on primary PCI. This can however be only delivered by a cardiologist in a highly specialised invasive cardiology hospital unit. Taking into consideration the above reasoning, strict aspiration/reservation values for P2 speciality and time-to-treatment values were assigned.

### Numerical results: EMS dispatching problem

The approach proposed in this paper is tested in simulations. For the test setup we took 41 real-life acute-state cardiac patients from the NEMSIS Data Set. This section presents the simulation results applied to the Problem 1, i.e. EMS Dispatching Problem. For testing purposes we assumed that 45 ambulances are available to respond to calls, since queuing models are not considered in the scope of current work. Time-to-arrival for each ambulance to each patient was chosen randomly from uniform distribution $t_p^a \in [6;200]$ min and speciality of the same from uniform distribution $s_a \in [0;1]$. Aspirations and reservations $a_{t_{EMS}}, r_{t_{EMS}}, a_{s_{EMS}}, r_{t_{EMS}}$ were assessed using expert knowledge, taking into account patient’s condition as described in the NEMSIS data set.

The optimisation results obtained through solving the Problem 1 are compared with results of two other goal functions—minimisation of total time-to-arrival and weighted sum aggregation, where two criteria are considered, i.e. minimisation of total time-to-arrival and maximisation of total speciality delivered. Those are given in (22) and (23)

22 $$\begin{aligned}{} & {} \text {min} \quad \sum _{p \in \mathcal {P}} t_p^1, \end{aligned}$$

23 $$\begin{aligned}{} & {} \text {min} \quad v_1 \sum _{p \in \mathcal {P}} t_p^1 - v_2 \sum _{p \in \mathcal {P}} s_p^1, \end{aligned}$$

where $v_1$ and $v_2$ are arbitrarily chosen weights for the weighted sum aggregation.

Due to space limitations we do not cite all the parameters and variables values, but we only give summary of the dispatch results obtained. An interested reader is welcome to reach out to the authors directly for all exact numerical results.

Results obtained by solving the EMS Dispatching Problem are shown in Table 3. We analyse the results on the following metrics:

- number of patients for whom resulting speciality and time-to-arrival were better than respective reservation value,
- greatest percentage gap over all patients between demanded reservation and the resulting value (for speciality and time-to-arrival),
- number of cases not meeting the reservation value by more than 10% (for speciality and time-to-arrival),
- total response time over all patients.

**Table 3:** EMS dispatching problem results.

Objective function	P1: EMS dispatching	(22)	(23), $v_1=1, v_2=1$	(23), $v_1=0.8, v_2=15$	(23), $v_1=15, v_2=0.8$
Speciality $\ge$ reservation	38	24	24	28	24
Time-to-arrival $\le$ reservation	35	33	33	33	33
Greatest gap (speciality)	9.49%	89.95%	89.95%	89.95%	89.95%
Greatest gap (time-to-arrival)	70.78%	99.64%	99.64%	99.64%	99.64%
No. of cases not meeting reservation by more than 10% (speciality)	0	11	11	7	11
No. of cases not meeting reservation by more than 10% (time-to-arrival)	4	6	6	7	6
Total response time [min]	951.54	502.98	502.98	512.31	502.98

As can be seen from the results, only when the proposed approach is applied (P1: EMS dispatching problem) speciality and time-to-arrival resulting from optimisation are much more often equal or better than the reservation. Such a behaviour is expected, since in benchmarking approaches reservation values are not taken into consideration. Yet, when the total response time is analysed, one may easily find that this metric is substantially larger in the proposed approach dispatch, than in the benchmarking strategies. This is because this metric is not controlled during optimisation process in the proposed approach. Aspirations and reservations are assigned individually for a given clinical condition, basing on medical knowledge. These values vary between clinical conditions. Taking aspirations/reservations into consideration in ambulance dispatch optimisation, allows for assigning ambulances in a way that they are met. As a result, the problem developed focuses on ambulance dispatch basing on patients’ condition and not simply treating all patients alike, as is done in benchmarking approaches. In that regard, some ambulances might be chosen that are further away from the calls (yet still within an acceptable distance), but offer better speciality. In that sense, the assignment result is more fit-for-purpose given the current operational state of the EMS system.

### Numerical results: ED dispatching problem

In this section we present numerical results of the proposed ED dispatching problem (P2) as obtained by optimising the test case. For this reason we took the very same patient cases as in the case study for P1, yet this time by taking into optimisation the values of $a_{t_{ED}}, r_{t_{ED}}, a_{s_{ED}}, r_{t_{ED}}$. They were estimated using the expert knowledge, taking into consideration patients’ condition as assessed by the EMS crew on scene and reported in the NEMSIS data set. Similarly to the P1 case study we compare the hospital dispatch obtained through solving the proposed problem with two other objective functions approaches, i.e.

24 $$\begin{aligned}{} & {} \text {min} \quad \sum _{p \in \mathcal {P}} t_p^2, \end{aligned}$$

25 $$\begin{aligned}{} & {} \text {min} \quad v_{12} \sum _{p \in \mathcal {P}} t_p^2 - v_{22} \sum _{p \in \mathcal {P}} s_p^2, \end{aligned}$$

where $v_{12}$ and $v_{22}$ are arbitrarily chosen weights for the weighted sum aggregation.

The ED dispatching problem proposed allows for differentiation of referential and non-referential hospitals. By design it helps the dispatcher to decide whether to dispatch the patient directly to a non-referential or referential unit, as well as to whether first dispatch them to a non-referential hospital and then to re-refer them to a referential unit. Of course such a re-referral comes at an increased time for the patient to reach their final ED destination.

To test the behaviour of the ED Dispatching Problem proposed under different decision situations (operating conditions) in terms of re-referrals, we test the approach under three scenarios, namely:

- *Scenario 1 (S1)*—both re-referrals and direct transport to referential hospitals are possible under *normal operating conditions*, where it is time-consuming to re-refer patient from a non-referential to a referential hospital.
- *Scenario 2 (S2)*—direct transports of patients to referential hospitals are not possible.
- *Scenario 3 (S3)*—both re-referrals and direct transport to referential hospitals possible, yet time of re-referral is assumed very little.

In the test case we assume existence of four hospitals in the considered EMS operating region—three non-referential (H1,H2,H3) and one referential (H4). Assumed speciality values on treating cardiovascular diseases of those hospitals are given in Table 4. Current capacity of EDs are assumed to be 20 patients for non-referential hospitals and 10 patients for the referential. Queuing in ED is not considered since it is deemed out of scope of this work. All time values, i.e. time-to-treatment of a given patient and times of re-referrals between hospitals were chosen randomly. Due to space limitations we do not cite these values in the paper, yet an interested reader is welcome to contact the authors and these values will be made available. For all testing cases we assumed $\eta _1=0.2$. This parameter is an arbitrary value, which says what percentage of non-referential hospital’s treatment capabilities is added for a patient, who is transferred further to a referential unit. It is only taken into consideration if the optimiser decides that a re-transfer between hospitals is needed, if not then it does not impact the speciality. If the value of the parameter is close to 1, the total speciality received by a re-transferred patient will be close to the sum of specialities offered by the non-referential and the referential units. Yielding specialities generally higher than required. This however will be coming at a large cost for time for re-transferring and not meeting the time reservation. By analogy, if it is close to 0 the speciality received will be close to the speciality offered by the referential facility and direct transfers will be preferred. This is because the non-referential unit would have very little impact on the overall treatment and re-transfer would worsen the time criterion. Having said the above, there is no clear linear relation between the number of re-transferred patients and the value of $\eta _1$. As a good practice and to reflect the clinical reality of re-transfers, we propose to keep it between 0.1 and 0.3, as always some sort of treatment will be applied (better stabilisation of condition or deepening of diagnostics).

**Table 4:** Assumed speciality of hospitals.

	Speciality
H1	0.18
H2	0.35
H3	0.56
H4	0.88

#### Scenario 1

In this section we give numerical results obtained by optimising the proposed ED Dispatching Problem under Scenario 1. These results are then compared with optimisation of goal functions (24) and (25). Those are shown in Table 5.

**Table 5:** ED dispatching problem results (S1).

Objective function	P2: ED dispatching	(24)	(25) $v_{12}=1, v_{22}=1$	(25) $v_{12}=0.8, v_{22}=15$	(25) $v_{12}=15, v_{22}=0.8$
Speciality $\ge$ reservation	37	18	18	22	18
Time-to-treatment $\le$ reservation	37	40	40	40	40
Greatest gap (speciality)	36.36%	77.50%	77.50%	77.50%	77.50%
Greatest gap (time-to-treatment)	41.19%	41.19%	41.19%	41.19%	41.19%
No. of cases not meeting reservation by more than 10% (speciality)	4	23	23	19	23
No. of cases not meeting reservation by more than 10% (time-to-treatment)	4	1	1	1	1
Total transfer time [min]	1554.50	742.64	742.64	752.22	742.64
Number of re-referrals	4	0	0	0	0
Number of direct transports to referential hospital	5	10	10	10	10

Similarly to the EMS dispatching problem (P1), speciality requirements (noted as being better than reservation levels) are met much more often as the result of the proposed P2 optimisation, than as the result of the benchmarking approaches. This comes at a slightly lower number of cases meeting the time requirements. This is compensated by higher speciality, where the greatest gap obtained by solving the P2 problem was 36.36% as opposed to 77.50% in other approaches.

The weights that we applied for solving the multi-criteria weighted sum aggregation (25) produced very similar results as opposed to each other. This is not an extensive list of weights and one should note that choosing different ones might produce different results. Yet, assignment of weights is a difficult task and is of little applicability in optimisation of dispatch of the EMS services, where decision must be made quickly and reliably.

#### Scenario 2

This section gives numerical results of test applied to Scenario 2, where direct transports to referential hospitals is not possible. In that sense, patients requiring specialised treatment will need to be first admitted to a non-referential ED and only then re-referred to a referential unit. This is a special scenario developed to analyse the impact of re-referrals applied instead of direct transport to referential hospitals. Results obtained are shown in Table 6.

**Table 6:** ED dispatching problem results (S2).

Objective function	P2: ED dispatching	(24)	(25) $v_{12}=1, v_{22}=1$	(25) $v_{12}=0.8, v_{22}=15$	(25) $v_{12}=15, w_{22}=0.8$
Speciality $\ge$ reservation	32	14	14	20	14
Time-to-treatment $\le$ reservation	38	40	40	40	40
Greatest gap (speciality)	48.57%	77.50%	77.50%	77.50%	77.50%
Greatest gap (time-to-treatment)	41.19%	41.19%	41.19%	41.19%	41.19%
No. of cases not meeting reservation by more than 10% (speciality)	9	27	27	21	27
No. of cases not meeting reservation by more than 10% (time-to-treatment)	3	1	1	1	1
Total transfer time [min]	1655.50	977.14	977.14	1015.80	977.14
Number of re-referrals	7	0	0	4	0
Number of direct transports to referential hospital	0	0	0	0	0

As can be seen forbidding direct transfers to referential units worsened the speciality results for all of the approaches shown. This is mostly due to the fact that re-referral is often costly in terms of time and therefore optimiser would opt for sacrificing the speciality in order to meet patients’ time requirements.

Thus, it is valid to conclude that re-referring is often not the best strategy and therefore, the dispatcher should always consider patients’ condition in the ED dispatching process to correctly direct the ambulance at the very moment of starting to transport the patient. Direct transports of patients to referential hospitals may greatly improve performance of the EMS services.

#### Scenario 3

Scenario 3 is a case, in which re-referral is much less time consuming than in normal operating conditions (S1), namely is one fourth of the re-referral time from S1. The purpose of testing under this scenario is to check if the proposed approach might be interesting to the dispatcher if re-referrals were not problematic from time perspective.

The results are shown in Table 7. As can be noted, from solving the proposed P2 ED Dispatching Problem many more re-referrals were obtained. However, changing the re-referral times did not change the results of other approaches considered. One may then conclude, that the P2 approach adapts itself better to changing decision environment from the approaches considered, while still being viable.

**Table 7:** ED dispatching problem results (S3).

Objective function	P2: ED dispatching	(24)	(25) $v_{12}=1, v_{22}=1$	(25) $v_{12}=0.8, v_{22}=15$	(25) $v_{12}=15, v_{22}=0.8$
Speciality $\ge$ reservation	40	18	18	22	18
Time-to-treatment $\le$ reservation	38	40	40	40	40
Greatest gap (speciality)	13.85%	77.50%	77.50%	77.50%	77.50%
Greatest gap (time-to-treatment)	75.25%	41.19%	41.19%	41.19%	41.19%
No. of cases not meeting reservation by more than 10% (speciality)	1	23	23	19	23
No. of cases not meeting reservation by more than 10% (time-to-treatment)	2	1	1	1	1
Total transfer time [min]	1410.80	742.64	742.64	751.22	742.64
Number of re-referrals	8	0	0	0	0
Number of direct transports to referential hospital	2	10	10	10	10

## Conclusions and discussion

The main goal of any system of emergency medical service is to provide timely and accurate medical support to patients in acute (often even life-threatening) condition. It is the task of the emergency medical dispatcher (together with the chief of the emergency crew) to correctly dispatch EMS crews (ambulances) to patients and patients onboard the ambulances to Emergency Departments in hospitals. This task is currently facilitated by the use of call triage and categorisations systems (eg. MPDS) and by means of patient transport protocols The condition of the patients varies greatly amongst them (basing on their clinical condition), and so varies the speciality in treating given kind of diseases between emergency system components. This is why treating all patients alike in the dispatch process, regardless of their clinical condition is not a desired assignment strategy. In some cases providing medical aid with wrong level of speciality might make the treatment less effective or even impossible.

In this paper we propose a multi-criteria optimisation approach to support both dispatching the EMS crews to patients and then patients to EDs. In that sense we develop a decision-support tool to be used by an emergency medical dispatcher in the dispatch process. The intent of the tool is to facilitate dispatcher’s decisions by providing them with Pareto-optimal recommendations on assignment decisions. The problems proposed consider patients’ requirements towards both time of getting medical support and the speciality level of this support. The requirements are expressed by means of aspiration and reservation values and assessed basing on patients’ health condition. We propose that this assessment can be performed by a properly trained medical personnel with the help of currently used MPDS system, and by integrating current transport protocols. Time and speciality requirements are not uniform across acute-state patients and depend greatly on their medical condition. We take this fact into consideration in our optimisation problems by optimising for both time and speciality requirements of each patient individually (on a per-patient basis), where aspirations/reservations calculated depend on their clinical condition. In that sense we can conclude that the results obtained by applying our method are more fit-for-purpose, and always consider the current operational state of the EMS system.

The proposed ED dispatching problem allows for optimising the decisions on whether to transport a given patient to non-referential facility or directly to a referential one, or to re-transfer them from a non-referential to a referential one. The decision is proposed by the optimiser taking into consideration all patients’ clinical condition, and the current operational state of the whole EMS system (availability of ambulances and hospital beds, speciality offered by available units, as well as time to reach the patient/ED). Thanks to optimising decisions considering this level flexibility, one can suspect that wide adoption of the system proposed, could possibly reduce offload delays in all types of hospitals. This is because patients will be transferred to destination hospitals, basing on their clinical needs. Therefore, it is likely that patients not requiring specialist care will be directed to non-referential units, and those requiring it to the referential one. Re-transferring will be done only when critically required. All of the above taking into consideration current hospital capabilities and delays.

In this work we propose also a framework to integrate the proposed problems into the current EMS/ED dispatching decision process, which outlines integration with already existing dispatch tools. The use of our method can potentially improve the performance of currently used techniques. Once the calls are categorised and triaged (job of MPDS), the method allows for identifying and assigning the most appropriate unit to work with a given patient, considering current operational state of the system as a whole. Similarly, the method can improve the use of current transport protocols by applying their guidelines in optimising for best ED (in terms of its address) to admit a given patient (in terms of exact hospital location), also considering current hospital capabilities. The approach is tested in simulations using real-life emergency cases stored in the NEMSIS data set over different decision environment scenarios.

In all scenarios tested, the proposed approach managed to find a dispatch that is better suited for the patients. This is measured by the number of patients receiving an emergency service being at least as good as the level of reservation—either towards time or towards the speciality of service. What is more, since we propose that the problems are scalarised using the Reference Point Method scalarisation, it is guaranteed that the dispatch obtained is always Pareto-optimal.

This research has also some limitations. First, we focused mostly on cardiological diseases for testing purposes. Yet, the problems proposed are generic enough, that the type of disease could easily be changed to any other. What is more, the problems could also be extended to introduce other speciality measures, towards other types of EMS service. These are however considered out of the scope of this paper.

As already stated, the method proposed is built on a per-patient basis and not on a per-incident basis. A question may then arise on how to handle EMS assignment for incidents with multiple patients. Our method is also capable of handling multiple patients in one event. When dealing with an accident with multiple patients, each one of them should be identified as needing help. And for each one of them we would have the time and speciality criterion assigned, with aspirations/reservations to each of them. In that sense, the dispatch of resources would be still in-line with the fit-for-purpose approach.

In case a mass event is present, it could simply not be possible to assign appropriate aspirations or reservations for EMS speciality needed, for each of the patients. In such a case we suggest to set the value of aspiration for speciality to 0.5 (middle of the range). Then, to assign reservation for the same to 0 (lower bound). However, we require that EMS arrive quickly and thus aspiration and reservation for time-to-arrival should be strict, e.g. reservation: 12 min, aspiration: 7 min. (depending on current operational state of the system, and on medical protocols). In that way the optimiser would aim to assign a unit, that can arrive at scene as soon as possible, with a slight preference towards more specialised ones.

Secondly, as in our approach the dispatching is not based solely on the time criterion, time-based EMS quality assurance Key Performance Indicators (KPIs) should possibly be adjusted. Considering a certain percentage of calls to be served within a nationally defined time threshold might no longer be appropriate. We propose to measure the EMS performance by the percentage of all criteria (time/speciality) which are at least as good as their reservation and compare it with a derived threshold. This however is to be applied at a legislative level.

In the optimisation problems we do not consider queuing, assuming that always at least as many EMS/ED units are available as the number of patients they should serve. Queuing can be considered as further research objective. Another research possibility is to apply the approach proposed into real-life emergency medical system dispatching.

Despite the limitations, the proposed approach proves to be an interesting dispatch strategy for EMS/ED dispatch. Differentiation of patients basing on their medical condition allows to better distribute limited EMS/ED resources in order to better suit patients’ needs. What is more, the proposed approach allows for consideration of both direct transports of patients to referential hospitals and re-referrals from non-referential units. In that sense, the approach gives more flexibility and allows for broader optimisation of dispatch decisions. All things considered, such an approach might possibly enhance patients’ survival rate in emergencies.

## Data Availability

This work is purely conceptual, aiming at the development of mathematical optimisation tool together with an EMS/ED dispatching framework. Neither did the research produce any data, nor it analysed any existing data. Case study was performed only to show the tool in action. For this, real-life emergency cases from the NEMSIS data set was used. Its correct references, where all relevant information can be found, have been cited in the manuscript. Aspirations/reservations values associated with patients’ condition were estimated using expert knowledge and were not fully given in the manuscript due to space and scope limitations. However, an illustrative example of such an estimation has been given in the manuscript. Speciality and time values were taken at random, wherever given in the manuscript. Nevertheless, an interested reader is encouraged to contact the corresponding author, shall they require all the data used in the case study. The corresponding author will be happy to make all case study data available upon request. The data sets used and/or analysed during the current study are available from the corresponding author on reasonable request.
